# Impact of the Use of Different Diagnostic Criteria in the Prevalence of Dyslipidemia in Pregnant Women

**DOI:** 10.5935/abc.20170070

**Published:** 2017-07

**Authors:** Alina Coutinho Rodrigues Feitosa, Luciana Tedgue Barreto, Isabela Matos da Silva, Felipe Freire da Silva, Gilson Soares Feitosa Filho

**Affiliations:** 1 Maternidade Professor José Maria de Magalhães Neto, Salvador, BA - Brazil; 2 Hospital Santa Isabel da Santa Casa de Misericórdia da Bahia, Salvador, BA - Brazil; 3 Escola Bahiana de Medicina e Saúde Pública, Salvador, BA - Brazil

**Keywords:** Dyslipidemias / diagnosis, Pregnancy / high-risk, Pregnancy Complications, Lipids, Prevalence

## Abstract

**Background:**

There is a physiologic elevation of total cholesterol (TC) and triglycerides (TG) during pregnancy. Some authors define dyslipidemia (DLP) in pregnant women when TC, LDL and TG concentrations are above the 95th percentile (p95%) and HDL concentration is below the 5th percentile (P5%) for gestational age (GA).

**Objective:**

To compare the prevalence of DLP in pregnant women using percentiles criteria with the V Brazilian Guidelines on Dyslipidemia and the association with maternal and fetal outcomes.

**Results:**

Pregnant women with high-risk conditions, aged 18-50 years, and at least one lipid profile during pregnancy was classified as the presence of DLP by two diagnostic criteria. Clinical and laboratorial data of mothers and newborns were evaluated.

**Conclusion:**

433 pregnant women aged 32.9 ± 6.5 years were studied. Most (54.6%) had lipid profile collected during third trimester. The prevalence of any lipid abnormalities according to the criteria of the National Guidelines was 83.8%: TC ≥ 200 mg/dL was found in 49.9%; LDL ≥ 160 mg/dL, in 14.3%, HDL ≤ 50 mg/dL in 44.4% and TG ≥ 150 mg/dL in 65.3%. Any changes of lipid according to percentiles criteria was found in 19.6%: elevation above the P95% for TC was found in 0.7%; for LDL, 1.7%; for TG 6.4% and HDL lower than the P5% in 13%. The frequency of comorbidity: hypertension, diabetes, smoking, obesity and preeclampsia was similar among pregnant women when DLP was compared by both criteria.

**Conclusion:**

The prevalence of DLP during pregnancy varies significantly depending on the criteria used, however none demonstrated superiority in association with comorbidities.

## Introdutcion

Lipids and lipoproteins change in gestation due to interactions between genetic, energetic and hormonal factors. Gestational hyperlipidemia is physiological and results from increased insulin resistance, lipoprotein synthesis and lipolysis in adipose tissue that mobilize fats to serve as an energetic substrate for fetal growth.^1-4^

The majority of pregnant women presents an increase in triglycerides (TG) in the third trimester, high density lipoproteins (HDL) in the second half of gestation, and progressive increasing of intermediate and low density lipoproteins (IDL) (LDL) over the trimesters.^5^ In the last trimester, total cholesterol (TC) may increase by 25 to 50% and TG by 200 to 400%.^6-7^

Dyslipidemia (DPL) in pregnancy is characterized by TG and TC concentrations above the 95th percentile and HDLs below the 5th percentile^8^, and this definition differs from that used for adults according to the *Expert Panel on Detection, Evaluation, and Treatment of High Blood Cholesterol In Adults* (NCEP)^9^ used in the V Brazilian Directive. Several researchers evaluated the lipids in gestation using the criterion of percentiles^5;10-14^ and reference values per quarter were established.^2^

Gestational hyperlipidemia is associated with metabolic morbidities such as obesity^15-16^ and gestational diabetes^1;6^ and is a risk factor for acute pancreatitis^17^, preeclampsia^3,15,18,19^ and preterm birth.^18-20^ Hypertriglyceridemia at the end of gestation is associated with the development of DLP in the postpartum decades^2;8^ and the offspring is at greater risk of being born large for gestational age^21^ and having atherosclerosis in adult life.^22-25^

Although pregnancy is recognized as a potential cause of DLP,^26^ the lipid profile is not part of the routine obstetric exams.^27^ The lack of consensus regarding reference values per trimester, lack of standardization of the diagnostic criteria, lack of determination of risk groups and of evidences demonstrating the impact of DLP treatment on pregnancy limit the accomplishment of screening.

In a population of high-risk pregnant women, we compared the prevalence of DLP defined by the criteria of the V Brazilian Dyslipidemia Directive with the specific criteria for pregnancy. We also evaluated the agreement between the criteria and the relationship between maternal lipid profile and maternal-fetal outcomes.

## Methods

### Study population

The population of the study was pregnant with an age between 18 and 50 years old, accompanied at the outpatient clinic of endocrine diseases during the gestation of the Maternity Professor José Maria de Magalhães Neto (MPJMMN) of Santa Casa da Bahia between April 2010 and March 2014. Those who had at least one lipid profile evaluation during gestation analyzed in a single laboratory and delivery in the MPJMMN. Demographic, clinical, obstetric, laboratorial, and labor data were obtained from medical records. The study was approved by the ethics and local research committee.

### Measurement of lipids

Blood samples were collected after a fast of 12-hour. Plasma concentrations of total cholesterol, HDLc and TG were determined by the automated colorimetric enzymatic method and LDL cholesterol was measured by the automated kinetic method. In pregnant women with more than one lipidogram collected during pregnancy, the first examination was considered for analysis. Less than half had more than one lipidogram in gestation. They made, respectively, two, three and four lipidograms during pregnancy: 109 (25.2%), 31 (7.2%) and 6 (1.4%). The gestational age (GA) at weeks at the time of blood collection was obtained from estimates of gestational age during the first ultrasound (USG). In patients in whom the first USG was not available, GA was estimated at the date of collection of the lipidogram by the date of the last menstrual period.

### Definition of dyslipidemia in pregnancy

The prevalence of dyslipidemia was evaluated considering two definitions:

1. "Percentile criteria" when there was elevation of TC, LDL-c and TG concentrations above the 95th percentile and HDL-c levels below the 5th percentile for gestational age.^5^ Normal values in pregnant women in the first, second and third gestational trimesters were obtained from the study of Piechota W and col^2^ and are available in table 1.

**Table 1:** Percentiles 95 for TC, LDLc and TG and 5 for HDLc in mg/dl according to Piechota and Staszekski^2^

Period	TC (mg/dl)	LDLc (mg/dl)	HDLc (mg/dl)	TG (mg/dl)
Out of pregnancy	251	167	34	171
1º Quarter	277	186	35	175
2º Quarter	319	217	42	254
3º Quarter	380	250	40	414

TC: total cholesterol; LDL: low density lipoproteins; HDL: high density lipoproteins; TG: triglycerides.
2. "Criteria of the V Brazilian Dyslipidemia Guideline" when TC, LDL and TG were, respectively, higher than 200 mg/dl, 160 mg/dl and 150 mg/dl, HDL-c, lower than 50 mg/dl.^28^ It was characterized as a carrier of dyslipidemia in pregnant women with at least one lipid fraction being altered.

### Maternal weight gain

Weight gain by the pregnant woman was categorized as below, in or above the target as recommended by the *Institute of Medicine*,^29^ which guides different weight gain intervals according to pregestational BMI.

### Neonatal outcomes

Neonates were classified as small for gestational age (SGA) when birth weight was below the 10th percentile and large for gestational age (LGA) when the weight was above the 90th percentile for gestational age and gender. The reference used was the Brazilian population of Pedreira et al.^30^ (2011).

Prematurity was defined as gestational age at birth between 24 and 36 weeks and 06 days of gestation. Preterm neonates were classified according to severity of prematurity in: preterm (< 31 weeks), preterm (31-33 weeks and 6 days) and preterm (4 to 36 weeks and 6 days) . Gestation dating was established through the first USG and somatic Capurro. In cases in which the first USG was not available (0.81%), GA was estimated at birth by LMP (Last Menstrual Period) and somatic Capurro.

### Statistical analysis

The data were analyzed to characterize the distribution normality by the Kolmogorov-Smirnov test. Continuous variables with normal distribution were presented as mean ± standard deviation and, for non-normal distributions, as median and interquartile range. Categorical variables were reported in absolute and percentage values. The subjects were categorized as having dyslipidemia for each of the two criteria and the statistical differences between continuous variables were obtained by means of the unpaired Student t test when the variables had normal distribution or by the *Mann-Whitney* U when they had a non-parametric distribution. The association between laboratory data of lipids and fractions, whose distribution was non-normal, with clinical variables: gestational age at birth, newborn weight and maternal weight gain were investigated using *Spearman*'s correlation tests. The concordance between the two criteria defining dyslipidemia was evaluated by the Kappa index. ROC curves were created, two in which the test variable was the percentile dyslipidemia criterion (curves A and B) and two (curves C and D) with the guideline criterion to determine the accuracy in predicting dichotomous weight outcomes of the neonate above the 90% percentile and gestational age at birth of 37 weeks or less. We calculated the area under the curve and the 95% confidence interval. Value of p < 0.05 was considered statistically significant. All analyzes were performed in the SPSS version 13 program.

## Results

A total of 433 pregnant women with a mean age of 32.9 ± 6.4 years and mean gestational age of 24.8 ± 7.6 weeks were evaluated. The main reason for referral to the outpatient clinic for endocrine diseases during pregnancy was diabetes, which represented 84.8% of the cases, thyroid diseases accounted for 6.9% of referrals and 43.2% were hypertensive. The clinical and demographic characteristics of the population are shown in table 2.

**Table 2:** Clinical and demographic characteristics of the general population categorized according to the presence of any lipidic alteration according to the percentiles criteria and the Brazilian V guideline (n = 433, results expressed as mean, standard deviation, median and interquartile range)

	Generaln = 433	Dyslipidemia Percentile criteria n = 85	Dyslipidemia Guideline criteria n = 363	No data	p
Age§	32,9 ± 6,5 33,1 (28,5–37,9)	31,9 ± 6,4 32,0 (27,6–37,5)	32,9 ± 6,4 33,1 (28,7–37,9)	0	0,1
First time mother(%)	24,7%	31,0%	25,7%	44	0,19
N by quarter (1º, 2º e 3º)	23/173/237 5,4/40,0/54,6%	4/34/47 4,7/40,0/55,3%	14/130/219 3,9/35,8/60,3%	0	
BMI pre-gestacional§ (kg/m2)	30,1 ± 6,5 29,7 (25,4–34,2)	29,0 ± 6,9 27,4 (23,4–32,5)	30,3 ± 6,5 30,1 (25,9–34,3)	108	0,09
Previous SAH (%)	167 (43,2%)	27 (38,6%)	141 (43,4%)	46	0,41
DM (%)	323 (84,8%)	57 (82,6%)	275 (85,7%)	52	0,39
Previous DLP (%)	80 (21,0%)	19 (27,9%)	69 (21,6%)	52	0,1
Smoking(%)	9 (2,4%)	3 (4,4%)	8 (2,5%)	51	0,25
Previous preeclampsia (%)	42 (14,0%)	7(14,9%)	36 (1,4%)	133	0,86
Previous preterm birth (%)	89 (20,6%)	18 (21,2%)	70 (19,3%)	0	0,58
Delivery mode (vaginal)	152 (35,1%)	31 (36,5%)	133 (36,6%)	0	0,92
TC (mg/dl) ¶	204,0 ± 83,1 199,0 (169,0–229,0)	212,8 ± 167,9 190,0 (151,0–235,0)	211,2 ± 88,2 206,1 (175,0–235,3)	6	0,005
LDLc (mg/dl) ¶	109,7 ± 42,8 105,0 (81,2–131,0)	115,2 ± 61,1 104,0 (71,0–151,0)	114,9 ± 43,6 111,0 (85,0–136,0)	22	0,22
HDLc (mg/dl) ¶	55,2 ± 15,1 54,0 (45,0–63,2)	40,9 ± 13,0 39,0 (35,0–45,8)	53,7 ± 15,4 51,0 (44,0–62,8)	11	< 0,0001
TG (mg/dl) ¶	199,9 ± 176 176,0 (136,0–229,0)	296,6 ± 664,2 224,0 (152,0–272,0)	215,8 ± 327,8 191,0 (152,0–237,0)	12	0,002
GA in childbirth (weeks) ¶	37,5 ± 3,0 38,0 (37,0–39,0)	37,4 ± 2,6 38,0 (37,0–39,0)	37,6 ± 2,9 38,0 (37,0–39,0)	0	0,15
Newborn weight (g) ¶	3187 ± 8523325 (2794–3677)	3183 ± 7923335 (2785–3695)	3195 ± 8063325 (2812–3686)	0	0,94
Glycated hemoglobin (g/dl) ¶	6,3 ± 1,7 6,0 (5,3–7,2)	6,7 ± 1,9 6,5 (5,4–7,9)	6,3 ± 1,6 6,0 (5,3–7,1)	141	0,09
Weight gain (kg) §	8,8 ± 8,3 8,6 (3,7–13,3)	7,9 ± 8,2 8,3 (1,1–14,2)	8,6 ± 8, 28,7 (3,6–13,6)	136	0,46
BMI adequacy B/I/A*	112/85/97 38,1/28,9/33	22/12/16 44,0/24,0/32%	92/69/84 37,6/28,2/34,3	139	

BMI: body mass index; hypertension: systemic arterial hypertension; DM: diabetes mellitus; DLP: dyslipidemia; GA: gestational age at delivery. TC: total cholesterol; LDL: low density lipoproteins; HDL: high density lipoproteins; TG: triglycerides.

* Adequacy of weight according to Institutes of Medicine (IOM): B low, I ideal and A high. For the p values: the groups were compared according to the presence of dyslipidemia considering the two criteria, when the distribution was normal, the Student’s t test was used; When the non-parametric distribution was used, the Mann Whitney U test was used.

**Figure 1:**
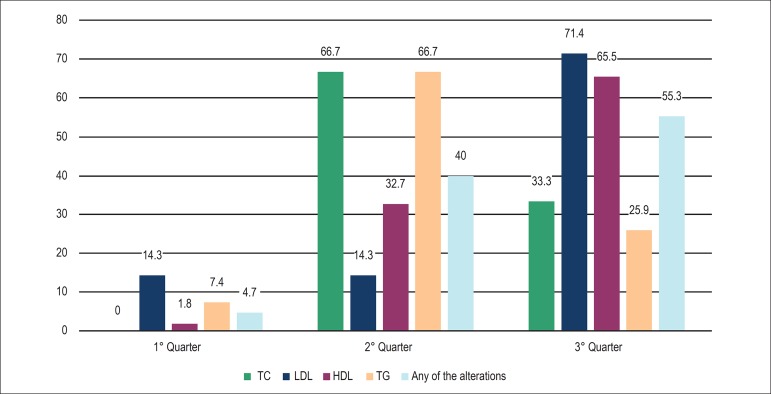
Prevalence of dyslipidemia according to percentile criteria. TC: total cholesterol; LDL: low density lipoproteins; HDL: high density lipoproteins; TG: triglycerides.

**Figure 2:**
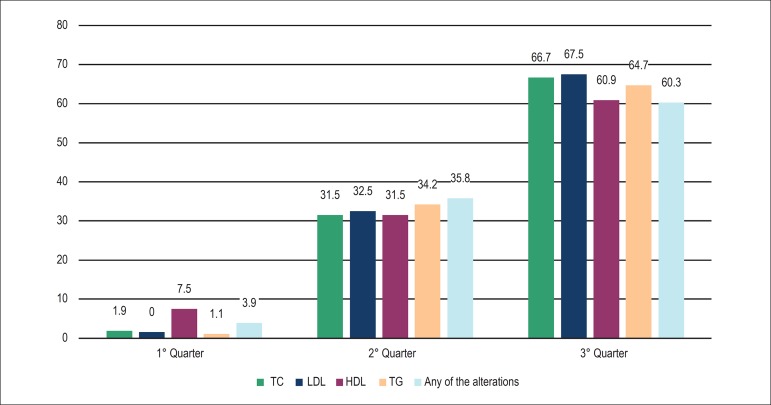
Prevalence of dyslipidemia according to guideline criteria. TC: total cholesterol; LDL: low density lipoproteins; HDL: high density lipoproteins; TG: triglycerides.

Dyslipidemia due to elevation of TC, LDLc and TG or reduction of HDLc was 4,3 times more frequent when dyslipidemia was considered by the V Dyslipidemia Guideline criterion in relation to percentile criterion (83.8 vs 19.6%). The 85 cases of dyslipidemia identified by the percentile criterion were among the 363 cases identified by the V guideline criterion. The most commonly found lipidic alteration was the reduction of HDLc, according to the criterion of percentiles and elevation of triglycerides, according to the criterion of V guideline (table 3). In addition, there was an increase in the frequency of any of the lipid changes with the progression of the quarters (figures 1 and 2).

**Table 3:** Prevalence of dyslipidemia according to the two criteria (n = 433)

Cholesterol and fractions	Percentile criteria General prevalence n (%) Prevalence per quarter n (%)	Guideline criteria General prevalence n (%) Prevalence per quarter n (%)	No data
TC	3 (0,7) 0 (0); 2 (66,7); 1 (33,3)	213 (49,9) 4 (1,9); 67 (31,5);142 (66,7)	6
LDL	7 (1,7) 1(14,3); 1(14,3); 5(71,4)	40 (11) 0 (0); 13 (32,5); 27 (67,5)	22
HDL	55 (13) 1 (1,8); 18 (32,7); 36 (65,5)	161 (44,4) 12 (7,5); 51 (31,5); 98 (60,9)	11
TG	27 (6,4) 2 (7,4); 18 (66,7); 7 (25,9)	275 (65,3) 3 (1,1); 94 (34,2); 178 (64,7)	12
Any lipidic alteration	85 (19,6) 4 (4,7); 34 (40); 47 (55,3)	363 (83,8) 14 (3,9); 130 (35,8); 219 (60,3)	22
All lipidic alteration	0 (0)	13 (3,6) 0(0); 2(15,4); 13(84,6)	22

TC: total cholesterol; LDL: low density lipoproteins; HDL: high density lipoproteins; TG: triglycerides.

The frequency of comorbidities SAH, DM, smoking, obesity and previous preeclampsia was similar when compared to pregnant women without dyslipidemia by any of the criteria. p = 0.005) and HDLc (39 vs 51 mg/dl, p = < 0.0001) were lower in patients with dyslipidemia by the criterion of percentiles, while the TG concentration was significantly higher (224 vs. 191 mg/dl, p = 0.002). There were no correlations between cholesterol and fractions with gestational age at birth and neonatal weight. Total and LDL cholesterol were inversely related to maternal weight gain (r = -0.173, p 0.003 and r = -0.177, p 0.003, respectively).

**Figure 3:**
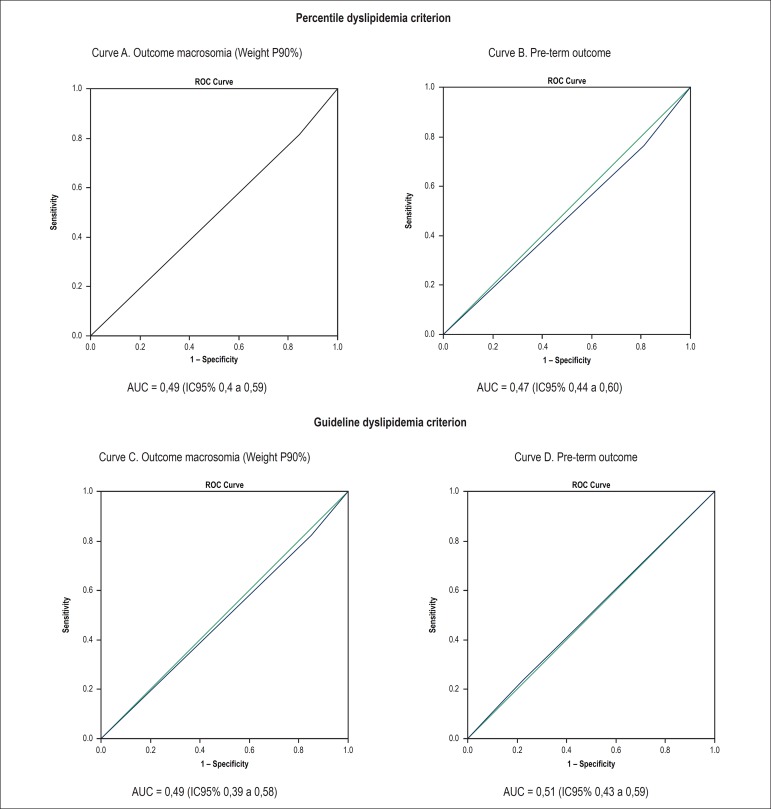
ROC curves of fetal outcomes and dyslipidemia by Percentile and Guideline criteria.

The agreement between the two criteria defining dyslipidemia was 0.09 (Kappa). All pregnant women with dyslipidemia according to percentile criteria were included in the guideline criteria. However, 80.2% of the women without dyslipidemia according to percentile criteria were dyslipidemic under Brazilian Guideline. The area under the curve (AUC) ROC for birth weight and gestational age revealed a lack of accuracy of both dyslipidemia criteria to predictthe risk of macrosomia and prematurity: dyslipidemia according to the National Guideline criteria, AUC 0.49 (95% CI 0%, 39 to 0.58) for birth weight and 0.51 (95% CI 0.43 to 0.59) for gestational age at birth; Dyslipidemia by percentile criterion, AUC 0.49 (95% CI 0.4 to 0.59) for birth weight and 0.47 (95% CI 0.44 to 0.60) for gestational age at birth, according to figure 3.

## Discussion

The present study revealed that, in a population of pregnant women with high gestational risk, the frequency of diagnosis of dyslipidemia according to the V guideline criterion was higher than that identified by the percentile criterion and that none of the criteria was able to predict risk of macrosomia and prematurity in the offspring of affected pregnant women. These findings show the impact of using different criteria to determine the prevalence of the same disease. However, the lack of association between the presence of dyslipidemia and fetal outcomes raises questions about the clinical importance of the detection of dyslipidemia during pregnancy by the criteria evaluated in this study.

The motivation to compare two diagnostic criteria for the definition of dyslipidemia during gestation was the lack of common understanding on the best way to diagnose dyslipidemia during pregnancy. The agreement between the two criteria was poor and this explained the significant difference in prevalence.

The adoption of a diagnostic strategy with the institution of cut-off points and defining criteria of a disease is not a simple task. In pathologies in which continuous variables such as lipids, blood pressure and blood glucose are used for diagnosis, choosing the best cut-off point to determine the disease is often difficult.^31^ One of the strategies used to establish cut-off points is the frequency-based statistical definition and distribution of the variable in the population. However, the determination of the optimal cut-off point for a diagnosis in the case of measurement of continuous variables depends on the finding of a value that maintains the balance between the medical, social and economic costs of making the diagnosis in people without substantial risk of adverse effects of one disease and not to diagnose those at real risk of disease damage.^32^ Establishing the diagnosis of a disease by statistical definition does not always reveal the association with risk and thus the value of the diagnosis. Glucose cutoff points for the diagnosis of diabetes, for example, were justified by the association with the dramatic increase in the prevalence of microvascular complications considered specific for diabetes^33^ which was not determined for lipids and poor fetal maternal outcomes.

The use of the criterion for the definition of dyslipidemia in adults resulted in a prevalence 4.3 times higher than that found by the criterion of specific percentiles for pregnant women. However, the frequency of comorbidities SH, DM and previous preeclampsia was similar in pregnant women with dyslipidemia by any of the criteria, when compared to those without dyslipidemia, and in the studied population there was no superiority of one of the criteria to identify pregnant women at greater risk . The areas under the ROC curve revealed a lack of accuracy in any of the criteria to establish the highest risk of macrosomia and prematurity. It is known that hypertriglyceridemia is associated with gestational diabetes and preeclampsia^15^ but it is unknown whether the strength of association with morbidities differs according to the criterion for dyslipidemia, however the present study revealed no association with both criteria. Using different criteria to diagnose the same disease, the result was the meeting of different prevalences that could have generated additional investigations, costs and unnecessary therapies. The similar proportion of comorbidities can be explained by the homogeneity of the population of the present study, consisting of pregnant women with high risk pathologies in which almost 90% were carriers of diabetes.

The present study revealed that the prevalence of dyslipidemia increased during the quarters when the criterion of V guideline was used. This finding is compatible with the physiological behavior of gestational hyperlipidemia and has already been demonstrated in several studies.^34;35^ However, the frequency of lipid changes was not progressive with the progression from gestation to cholesterol and TG when the criterion of percentiles was used. It is possible that the limited number of patients with alterations in these lipid fractions favored chance and not demonstrated the physiological behavior of the lipids for the percentiles criterion. However, when analyzing dyslipidemia for any lipid alteration, increasing the sample size for each lipid fraction, we found that, for both criteria, dyslipidemia was more frequent with the advancement of the quarters.

Controversies and questions about different definitions for the same disease are international. Regarding dyslipidemia in pregnant women, the scientific community claims for attention and research on lipid metabolism during gestation.^3^ The fact that there is no standardization of the ideal criterion may result in the doctor who assists the pregnant woman choosing any of the criteria without the benefit of the use being demonstrated. In the absence of evidence of cut-off points that identify the possible risk(s), it seems reasonable to use the percentiles definition strategy based on the frequency of lipid distribution during pregnancy. However, there are problems that limit the use of percentile criteria: it is necessary to have a reference table for pregnancy lipids categorized by quarter to establish cut points for each lipid and there is no established and internationally accepted standard. There are few studies that report reference values per quarter.^2;34;36^ and none in Brazil, to our knowledge. There are several Brazilian publications that demonstrate associations between lipids and BMI,^37^ depressive symptoms,^38^ changes in pressure,^39^ risk of gestational diabetes,^40^ without, however, using cut-off points to determine the increase in morbidities or specific risks for dyslipidemia during pregnancy. The present study demonstrated that pregnant women with dyslipidemia defined by guideline criteria had higher TC and HDL and lower TG concentrations than pregnant women with dyslipidemia according to percentile criteria, suggesting that the guideline criterion selects cases of greater severity in relation to dyslipidemia . However, the study did not show superiority of any of the criteria in relation to the association with other maternal or fetal morbidities raising the question of why to diagnose a disease that does not modify maternal-fetal clinical outcomes. These findings, however, should be confirmed with a higher number of pregnant women and in low-risk pregnancies to define the importance of dyslipidemia during pregnancy and which diagnostic criteria to use.

This task presents, however, some limitations. It is a unicentric study with pregnant women from the state of Bahia. The sample is presumed to be mixed, however, it is known that important population differences occur according to the region of the country and Bahia is the state with the highest percentage of African contribution to ancestry.^41^ While the impact of ethnic/racial differences in the relation between dyslipidemia and rates of cardiovascular disease lack determination, non-hispanic blacks and whites are less likely to have dyslipidemia than Asian and Mexican Americans,^42^ and we do not know if the same is true for the Brazilian population. The results of the present study therefore do not allow national or international generalization and the absence of association with clinical outcomes may have been a result of racial influence and/or limited sample size.

Most of the pregnant women had only one determination of the lipidogram and we know that the lipid fractions, especially the concentration of triglycerides, undergo significant changes depending on diet, exercise and intra and inter-laboratory variations. Despite recognizing the possibility of the influence of an isolated determination on the reduction of the robustness of the findings, a significant portion of the observational studies investigating associations between dyslipidemia and outcomes, do it with only determination, so that our study does not differ from the method commonly used in research in this area. All measurements were made in a 12-hour fasting period, which is also the most used method to determine the lipidogram and to investigate the outcomes related to dyslipidemia.

The cross-sectional nature of the study with data collection in medical records is a limitation, as far as confounding factors could have been neglected. Lack of recognition and appropriate assessment of the influence of confounding factors possibly interferes with results. Most of the pregnant women investigated were diabetic and obese, morbidities associated with dyslipidemia, and the independent contribution of each morbidity in the findings was not established. While the limitation in establishing the causal relationship due to lack of evidence of the temporal sequence between exposure to the risk factor and disease development is a recognized disadvantage of cross-sectional studies, this type of study is important for calculating disease prevalence, the main focus of the present study.

## Conclusion

The prevalence of dyslipidemia assessed by the V Brazilian guideline for adult dyslipidemia was higher than the prevalence identified by the criterion of the specific percentiles of pregnancy without, however, showing superiority in the association with morbidities.
